# Gender differences in presentation, management, and outcomes among Egyptian patients with acute coronary syndrome: a single-centre registry

**DOI:** 10.1186/s12872-024-03996-8

**Published:** 2024-07-16

**Authors:** Mohammed Elbarbary, Hazem K. Shalaby, Salma M. Elshokafy, Mohamed A. Khalil

**Affiliations:** Department of Cardiovascular Medicine, Faculty of Medicine, Tanta University, Tanta, Egypt

**Keywords:** Gender differences, Women, Acute coronary syndrome, Hospitalization and mortality, Outcomes, Low middle-income countries

## Abstract

**Background:**

Despite a significant rise in cardiovascular disease (CVD)-related mortality in low- and middle-income countries (LMICs), data are scarce regarding the quality of care provided, particularly for women.

**Methods:**

This is a prospective observational, cross-sectional study. Acute coronary syndrome (ACS) patients presented to the Cardiology Department at Tanta University, Egypt, between September 1, 2023, and December 31, 2023, were enrolled. The study assessed gender disparities by comparing men and women regarding presentation, management, and major adverse cardiovascular events (MACE) occurrence during hospitalization and 30 days after discharge.

**Results:**

A total of 400 ACS patients were included, with 29.5% being women. Women were comparatively older (59 ± 9 years vs. 55 ± 13 years), with a significantly higher prevalence of hypertension (70.3% vs. 47.5%) and diabetes (55% vs. 36.8%). Non-ST-segment elevation myocardial infarction (Non-STEMI) was more common in women (35.29% vs. 21%). Dyspnea was expressed by 34.4% of women (vs. 21.35% of men). Women were hospitalized later (9.29 h vs. 6.74 h). In-hospital outcomes were poorer for women with worse NYHA classes III and IV. Additionally, the odds ratio (OR) for in-hospital cardiac mortality was 0.303 (95% CI 0.103–0.893*)* for women compared to men. However, a one-month follow-up for MACE post-hospital discharge did not indicate significant gender differences.

**Conclusions:**

The current study suggests that women with ACS in Egypt exhibit a higher risk profile for CVD compared to men and tend to present later with atypical symptoms. Women additionally experience poorer in-hospital MACE and higher cardiac mortality. Therefore, increasing awareness about ACS syndrome and eliminating obstacles that delay hospital admission are imperative.

**Supplementary Information:**

The online version contains supplementary material available at 10.1186/s12872-024-03996-8.

## Introduction

Cardiovascular disease (CVD) is the primary cause of mortality among women globally, taking the lives of approximately 6 million individuals annually. Notably, women admitted to hospitals with acute coronary syndrome (ACS) often experience inferior outcomes compared to men [1, 2].

Studies, particularly from high-income countries (HICs), have revealed disparities in the treatment of ACS between men and women. Women are less likely to receive guideline-recommended pharmacotherapy and undergo invasive procedures like angiography and percutaneous coronary intervention (PCI) [3, 4]. Additionally, an increased risk of adverse clinical outcomes was observed for women with ACS undergoing an early invasive strategy and coronary revascularization compared with men [5].

Despite a significant rise in the prevalence of cardiovascular disease-related mortality in low- and middle-income countries (LMICs) [6] ; data are scarce regarding the quality of care provided for women [7], and only a few reports are available from specific regions, especially Africa [8]. According to the World Bank classification, Egypt falls within the lower-middle income category [9]. Although CVD accounted for 46.2% of the overall mortality in Egypt in 2017, studies evaluating gender differences in ACS in Egypt are limited [10].

This study aimed to identify gender disparities in the presentation, management patterns, and outcomes of ACS patients in Egypt. Such research is crucial for clinicians, cardiologists, and global health experts to improve awareness and guidelines about women’s heart health in Egypt and other LMICs.

## Methods

### Sample size

The sample size was determined using EPI Info (Centers for Disease Control and Prevention, Atlanta, GA, USA), version 7 [11]. Previous studies by Ibrahim et al. [12] and Mansour et al. [13] estimated the prevalence of ACS in Egypt to be approximately 8.3%. While using a 5% margin of error, a 99.9% confidence level, and a response distribution of 80%, the minimum representative sample size was initially estimated to be 330 patients. Still, this investigation increased the sample by 20% to accommodate any potential loss during the follow-up after hospital discharge. As a result, four hundred patients were enrolled.

### Study design

The present study is a prospective cross-sectional observational study conducted at the cardiovascular department of Tanta University Hospital, Gharbia Governorate, Egypt, between September 1, 2023, and December 31, 2023. The study was approved by the ethical committee at the Faculty of Medicine, Tanta University, Egypt, (Clinical Trial Number: 36264PR395/10/23).

### Inclusion criteria

The study included male and female patients aged > 18 who consecutively presented with ACS. ACS has been defined according to the guidelines established by the European Society of Cardiology (ESC) [14] and includes two distinct presentations: ST-segment elevation ACS (STEMI), characterized by acute chest pain with persistent ST-segment elevation lasting for more than 20 min, and Non-ST-segment elevation ACS (Non-STEMI). The latter refers to patients experiencing acute chest pain without ST-segment elevation on electrocardiogram (ECG) but exhibiting ECG changes such as transient ST segment elevation, persistent or transient ST segment depression, T wave inversion, flat T wave, pseudonormalization of T waves, or a normal ECG with positive troponin enzyme levels.

### Exclusion criteria

Patients who refused to give informed consent, those with insufficient medical records, patients who skipped follow-up appointments, and patients with severe morbidity affecting survival, such as cancer, were excluded.

### Data collection

It included a comprehensive history-taking process focused on age, smoking status, and family history, as well as a thorough evaluation of the complaint and presenting symptoms, including onset and duration.

Specific attention was placed on established risk factors, including dyslipidemia, diabetes mellitus, and hypertension. Dyslipidaemia is defined as having a history of lipid-lowering therapy or low-density lipoprotein (LDL) cholesterol > 70 mg/dl [15]. Hypertension is described as having a history of hypertension or systolic or diastolic blood pressure ≥ 140/90 mmHg [16]. Diabetes (type 1 or 2) is a history of diabetes or fasting plasma glucose > 126 mg/dl.

### Diagnostic procedures

A well-standardized 12-lead resting ECG was performed with particular emphasis on heart rate, cardiac rhythm, conduction abnormalities, ST segment deviation, and T-wave changes. Laboratory investigations comprised troponin I, complete blood counts, HbA1c, serum creatinine, and lipid profiles.

Bedside echocardiograms were performed following the American Society of Echocardiography recommendations [17] for assessing ejection fraction (EF) and resting wall motion abnormalities, mechanical complications, substantial valvular lesions, and other aberrant findings. Furthermore, the echocardiography was repeated on the discharge day morning.

### Treatment and follow-up

Medications, management strategies, and in-hospital courses were recorded. Coronary angiography and intervention were conducted for STEMI and high-risk non-STEMI patients following ESC guidelines [18]. Patients were cautiously observed for contrast-induced nephropathy (CIN) and significant bleeding. The latter is defined according to the Global Utilization of Streptokinase and Tissue Plasminogen Activator Criteria for Occluded Coronary Arteries [19].

Furthermore, this trial monitored every patient for 30 days post-hospital discharge, where follow-up visits were arranged once every two weeks at the outpatient clinic.

### Study outcomes

The primary outcomes are in-hospital and 30-day post-discharge cardiac mortality and other major adverse cardiovascular events (MACE) events, including reinfarction, heart failure (NHYA III/IV), cerebral stroke, and any unplanned readmission for heart failure and/or reinfarction. Cardiovascular mortality was used to calculate the composite of MACE per patient when accompanied by other MACE events.

### Data analysis

A descriptive analysis was performed using means and standard deviations for parametric tests and means and medians for non-parametric tests. The chi-square test was employed to assess the differences between groups based on categorical variables. At the same time, the unpaired t-test was used for continuous variables when the Komolgorov-Smirnov normality test confirmed a normal distribution and a significance level of *p* < 0.05 was established. The Mann-Whitney U test was applied for non-normally distributed data.

- The incidence rates of clinical outcomes were based on gender. The OR and confidence intervals (CI) were reported.
- Multivariate logistic regression identified potential predictors for MACE, considering *p* < 0.05 as significant.
- Kaplan-Meier curves and log-rank tests assessed 30-day MACE survival differences between men and women.
- Statistical analyses were performed online using DATAtab: Online Statistics Calculator, provided by DATAtab e.U. Graz, Austria. URL: https://datatab.net [20].

## Results

### Baseline characteristics (table 1)

**Table 1:** Baseline demographic and clinical characteristics of ACS patients

	Men		Women		*P* value	Total	
	*n* =	%	*n* =	%		*n* =	%
Age ^a^	55.68 ± 13.05		59.78 ± 9.75		0.001*	56.84 ± 12.25	
BMI (kg/m2) ^a^	30.52 ± 21.87		34.7 ± 20.48		0.149*	31.66 ± 21.55	
Type of ACS							
STEMI	222	79%	77	64.71%	0.003 **	299	74.75%
Non STEMI	59	21%	42	35.29%	0.003 **	101	25.25%
Marital status :							
Married	257	91.1%	96	81.3%	< 0.001 **	353	88.25%
Widow	17	6%	21	16.9%		37	9.25%
occupation							
Not employed	128	45.3%	90	76.2%	< 0.001 **	218	54.5%
Employed	88	31.4%	11	9.3%		99	24.75%
Retired	66	23.4%	17	14.4%		83	20.75%
Cardiovascular risk factors							
Currently smoke	175	62.28%	19	15.97%	< 0.001**	194	48.5%
hypertension	134	47.51%	83	70.33%	< 0.001 **	217	54.25%
DM	104	36.87%	65	55%	0.001 **	169	42.25%
Dyslipidemia	220	78%	84	71.18%	0.283 **	304	76%
FH of CAD	16	5.63%	8	6.9%	0.629 **	24	6%
Previous AMI	43	15.25%	9	7.62%	0.041 **	52	13%
Previous PCI	41	14.54	15	12.7%	0.635 **	56	14%
Previous CABG	2	0.5%	2	0.5%	0.361 **	4	1%
Previous CHF	13	4.6%	3	0.25%	0.336 **	16	4%
Previous iCVA	26	9.2%	9	7.6%	0.607 **	35	8.75%
Renal insufficiency	10	3.5%	3	2.5%	0.606 **	13	3.25%

^*a*^ *Results are expressed as mean ± standard deviation*

*Unpaired t-test

** chi-square test

*Abbreviations: BMI: body mass index; DM: diabetes mellitus; FH: family history; CAD: coronary artery disease; iCVA: ischemic cerebrovascular accident; AMI: acute myocardial infarction; CABG; coronary artery bypass grafting*

A total of 65 patients (49 men and 16 women) were excluded from the study because they had not completed their post-hospital discharge follow-up at our clinic, and therefore their 30-day outcomes were missed. Finally, this study enrolled 400 patients diagnosed with ACS. Women constituted 29.5% of the cohort. Men exhibited a higher prevalence of STEMI than women (79% vs. 64%; *p* = 0.003), whereas non-STEMI was more common among women (35.2% vs. 21%; *p* = 0.003). Women were older (59 ± 9 years vs. 55 ± 13 years, *p* = 0.001). The most represented age group for both genders was between 56 and 65 (see Supplementary Fig. 1, Additional File 1).

There was a statistically significant difference in marital status, with a notable percentage of widows among women (17.6% vs. 6.0%; p = < 0.001). Additionally, most women were unemployed (76.2% vs. 45.3%, p = < 0.001). In contrast to women, the prevalence of current smoking was notably higher among men (62.28% vs. 15.97%, *p* < 0.001).

Regarding clinical risk factors, the prevalence of hypertension was more significant among women (71.5% vs. 47.1%; *p* < 0.001), and the percentage of diabetic women was higher compared to men (54.6% vs. 37%, *p* = 0.001). Previous myocardial infarction (MI) was more common in men (15.25% vs. 7.69%, *p* = 0.041). Men have had previous PCI in a similar percentage to women (14,54% vs. 12.8%; *p* = 0.653).

### Presentation (table 2)

**Table 2:** Clinical presentation and ECG findings between the two groups:

variables	men		Women		*p* value	Total	
	*n*	%	*n*	%		*N*	100%
Clinical presentation:							
Angina pain	274	97.1%	110	93.2%	0.82**	384	96%
Dyspnea	60	21.35%	41	34.45%	0.006**	101	25.25%
Palpitation	12	4.25%	10	8.4%	0.073**	22	5.5%
Syncope	2	0.71%	1	0.85%	0.884**	3	0.75%
Killip class III	2	0.71%	4	3.39%	0.161**	6	1.5%
Killip class IV	2	0.71%	2	1.69%		4	1%
ST segment changes in ECG:							
Persistent ST Elevation	218	77.3%	74	62.71%	0.005**	292	73%
ST depression	47	16.67%	34	28.81%		81	20.25%
Transient ST elevation	6	2.13%	5	4.24%		11	2.75%
LBBB	10	3.55%	2	1.69%		12	3.06%
unremarkable ECG changes	1	0.35%	3	2.54%		4	1%
Cardiac rhythm :							
Heart rate	81.16 ± 16.56		87.54 ± 27.61		0.023*	83.01 ± 20.6	
Sinus rhythm	264	93.6%	104	88.13%	0.218**	368	92%
AF	6	2.12%	7	5.9%		13	3.25%
CHB	2	0.71%	2	1.6%		4	1%

^*a*^ *Results are expressed as mean ± standard deviation*

*Unpaired t-test;

** chi-square test

*Abbreviations: HB; heart block. CHB; complete heart block. AF; atrial fibrillation. V.Tach; ventricular tachycardia*

Anginal pain was the most common presenting symptom expressed by approximately 96% of the total patients, followed by dyspnea, which was more common in women (34.45% vs. 21.35% in men, *p* = 0.006). Palpitation was associated with ECG evidence of arrhythmias, especially atrial fibrillation, in 13 cases, while three actual syncope cases were encountered because of complete heart block (CHB).

Women exhibited a significantly higher heart rate than men (87.5 ± 27.6 vs. 81.1 ± 16.5, *p* = 0.023). Most patients (97.5%) were classified as Killip Class I and II, with 1.5% in Class III and 1% in Class IV, displaying no statistically significant difference. However, in STEMI subgroup analysis, more women presented in Killip classes III and IV than men (4.2% vs. 0.35%, *p* = 0.006).

The two groups had no statistically significant difference regarding laboratory results, including total cholesterol, LDL, HDL, on-admission creatinine, on-admission RBG, on-admission troponin, and glycated haemoglobin (see Supplementary Table 1, Additional File 2).

### In-hospital management/pharmacotherapy

Male patients received higher prescription rates for aspirin, ticagrelor, ACEIs, and statins compared to females. Over 70% of patients were given beta-blockers, and over 78% were given angiotensin-converting enzyme inhibitors or angiotensin receptor blockers (ACEI/ARBs) (see Supplementary Table 2, Additional File 3).

### In-hospital management/coronary intervention (table 3)

**Table 3:** Coronary angiography and its findings among the enrolled men and women:

Variable	men		women		*P* value	total		
	*n*	%	*n*	%		*n*	%	
Time from ACS to CCU (h) ^a^	6.74 ± 6.9		9.29 ± 10.9		0.001***	7.48 ± 8.3		
Door-to-balloon time (minutes) in pPCI cases ^b^	115.5 (45)		116 (75)		< 0.001***	115.7 ( 45)		
Length of hospital stay (days) ^a^	3.0 ± 0.6		3.1 ± 0.9		0.278*	3.0 ± 0.7		
LVEF (%) at discharge. ^a^	49.6 ± 8.1		49.46 ± 8.5		0.564*	49.56 ± 8.		
Coronary angiography done:								
STEMI subgroup	211	74.8%	70	59.3%	0.189**	281	70.25%	
Non-STEMI subgroup	31	10.9%	28	23.7%	0.156**	59	14.75%	
Access								
radial	92	32.6%	36	30.5%	0.954**	128		32%
femoral	150	62%	62	52.5%		212		53%
Culprit vessel :								
RCA	52	18.4%	19	16.1%	0.035**	71		17.75%
LCX	54	19.15%	24	20.3%		78		19.5%
LAD	134	47.5%	49	41.5%		183		45.5%
Coronary angiography diagnosis :								
Single vessel disease	106	72.6%	40	33.8%	0.755**	146		36.5%
Two vessel disease	85	30.1%	30	25.42%	0.313**	115		28.75%
Three vessel disease	49	17.3%	22	23.71%	0.663**	71		17.75%
MINOCA	2	0.7%	6	5%	0.003**	8		2%

^*a*^ *Results are expressed as mean ± standard deviation*

^*b*^ *Results are expressed as mean and median*

*Unpaired t-test

** chi-square test

*** Mann Whitney U test

*Abbreviations: ACS. acute coronary syndrome. CCU; cardiac care unit. STEMI; ST-segment elevation myocardial infarction. NSTEMI; Non-ST segment elevation myocardial infarction; PCI; Percutaneous coronary intervention; LM; left main artery. LAD. left anterior descending. RCA; right coronary artery. LCX; left circumflex. MINOCA; Myocardial infarction with no obstructive coronary artery disease*

The time between symptom onset and hospital admission was longer for women (mean = 9.29 h, median = 10.9 vs. 6.74 h, median = 6.9, *p* = 0.001). Additionally, in the STEMI subgroup undergoing primary PCI (pPCI), door-to-balloon time was longer in women compared to men (mean = 116 min, median = 75 min vs. mean = 115.5 min, median = 45 min, *p* = 0.01) (see Supplementary Fig. 2, Additional File 4).

An early invasive strategy was employed in 85% of all ACS patients. No gender variation was noted regarding reperfusion strategy, intervention access, culprit vessel, coronary anatomy complexity, number of stents, or procedural complications (see Supplementary Table 2, Additional File 3).

MI with no obstructive coronary artery disease (MINOCA) was more prevalent in women (6 cases vs. 2 cases, *p* = 0.003). Streptokinase was administered as thrombolytic therapy to 48 STEMI patients. Additionally, in 4.91% of cases, an urgent coronary artery bypass graft (CABG) was recommended, and temporary pacemaker implantation was performed in four cases with CHB.

### In-hospital outcomes (table 4)

**Table 4:** In-hospital course, MACE and 30-day outcomes between the two groups:

Variable	Men		women		Odds ratio		95% Confidence Intervals		*P* value
	*n*	%	*n*	%			Lower	Upper	
In-hospital course:									
Post PCI CIN	2	0.71%	1	0.85%	1.59		0.442	5.76	0.47
Blood transfusion	3	1%	4	3.38%	3.22		0.710	14.6	0.11
Major bleeding	1	0.35%	1	0.85%	2.37		0.147	38.3	0.53
Cardiogenic shock	4	1.42%	3	2.54%	1.79		0.395	8.13	0.44
In hospital MACE:									
Reinfarction	0	0. %	1	0.85%	7.13		0.288	176	0.124
NYHA class III or IV	22	7.8%	17	14.41%	1.96		1.00	3.85	0.047
Stroke	1	0.4%	0	0%	0.782		0.0316	19.3	0.515
Cardiac mortality	6	2.13%	8	6.78%	0.303		0.103	0.893	0.022
Composite	25	8.9%	23	19.3%	2.45		1.33	4.53	0.003
30-day MACE:									
Unplanned readmissions for Heart Failure	14	6.1%	7	8.4%		1.41	0.550	3.64	0.470
Unplanned Readmissions For Re-Infarction	3	1.3%	3	3.7%		2.83	0.560	14.3	0.189
Stroke	3	1.1%	2	1.7%		1.57	0.259	9.54	0.62
Cardiac mortality	9	3.6%	7	5.9%		1.70	0.630	4.57	0.291
Composite	27	9.6%	19	16.0%		1.79	0.951	3.36	0.068

*Abbreviations: PCI; precautious coronary intervention. CIN; contrast-induced nephropathy. NYH; New York Heart Association. MACE; major adverse cardiovascular events*

The duration of hospital stay revealed no significant difference (3.01 ± 0.62 days for men vs. 3.11 ± 0.9 for women; *p* = 0.278). Likewise, there were no statistically significant differences between the two genders as regards discharge EF%, post-PCI CIN, rate of blood transfusion, access site major bleeding, or cerebral stroke occurrence.

However, the in-hospital outcomes were poorer in women with higher NYHA classes III and IV rates. The OR for in-hospital cardiac mortality was 0.303 (95% confidence interval [CI] 0.103–0.893) for women vs. men. Conversely, the OR for the composite of in-hospital MACE was higher in women at 2.45 (95% confidence interval [CI] 1.33–4.53) compared to men.

### 30-day outcomes (table 4)

**Fig. 1:**
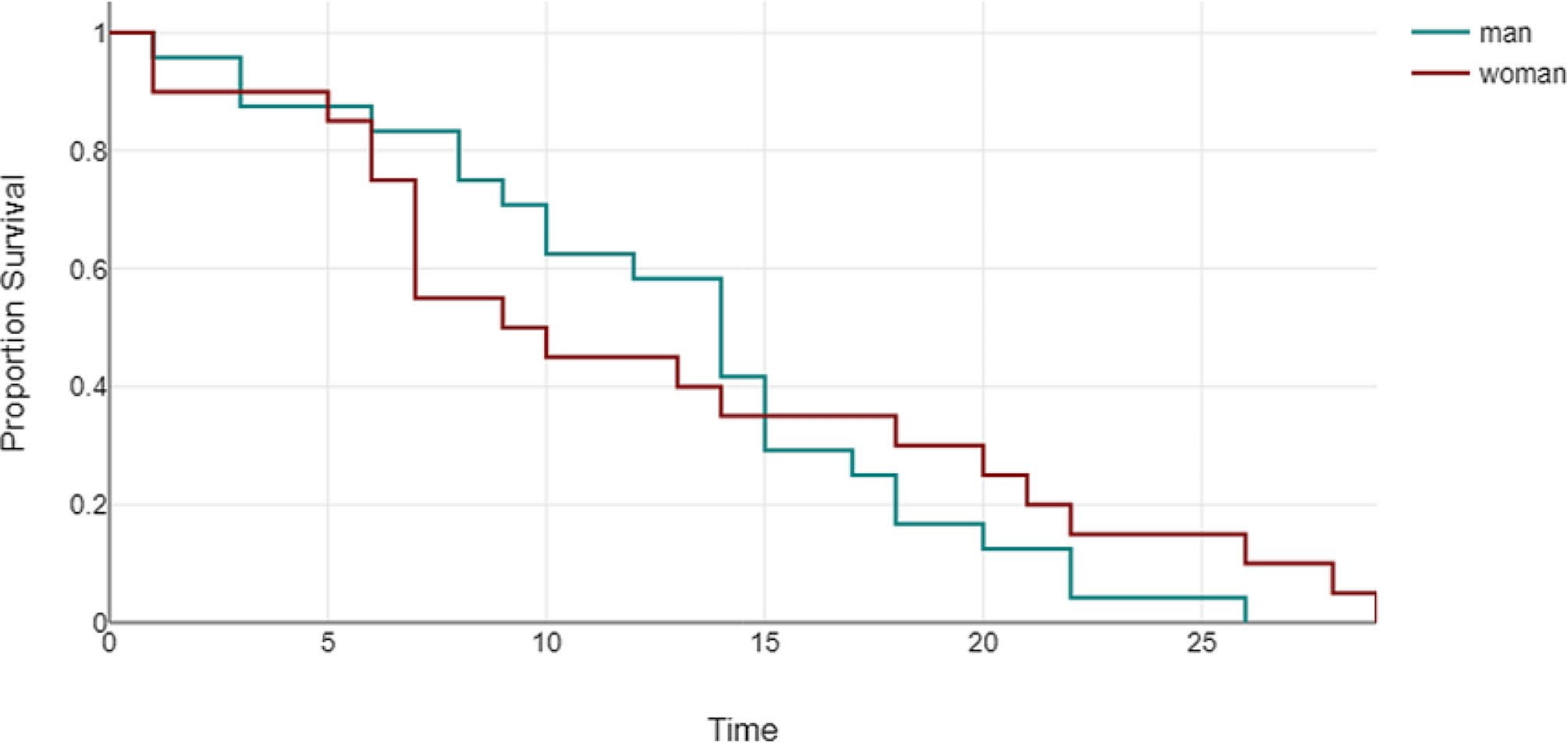
An extendedrank test the log-rank test exhibits no significant statistical difference between men and women regarding 30-day MACE events, P value = 0.66

A one-month duration of follow-up did not exhibit a significant difference between men and women regarding unplanned readmission for heart failure, reinfarction, stroke, or cardiac mortality, with no statistically significant findings observed between genders (*P* = 0.47, 0.189, 0.62, and 0.291, respectively). The log-rank test indicated no disparity between genders in the time distribution until MACE events occurred (*p* = 0.66) (Fig. 1).

A multivariable logistic regression model was constructed to identify potential in-hospital and 30-day MACE predictors. The results revealed that older age, female gender, renal impairment, atrial fibrillation, and impaired EF < 40% are potential independent predictors with respective *P*-values of (0.046, 0.015, 0.004, 0.002, and < 0.001) (Table 5).

**Table 5:** Univariate and multivariate regression analysis for predictors of all MACE events (both in hospital and 30-day):

Variable	Univariate analysis			Multivariate analysis		
	Odds Ratio	95% conf. interval	*P* value	Odds Ratio	95% conf. interval	*P* value
age	1.05	1.02–1.07	< 0.001	1.03	1–1.06	0.046
Gender - Women	2.19	1.27–3.78	0.005	2.48	1.19–5.16	0.015
Current smoking	2.24	1.28–3.93	0.005	0.83	0.4–1.74	0.624
HTN	1.32	0.77–2.27	0.31	0.71	0.33–1.5	0.367
DM	1.89	1.1–3.22	0.02	1.96	0.98–3.93	0.058
Renal impairment	4.16	1.39–12.42	0.011	6.8	1.84–25.2	0.004
Previous MI	0.93	0.41–2.08	0.856	1.2	0.46–3.13	0.707
AF	4.77	1.55–14.68	0.007	7.45	2.04–27.27	0.002
Three vessel CAD	1.92	1.01–3.67	0.048	1.49	0.66–3.39	0.339
EF < 40%	5.74	2.07–15.9	0.001	11	5.28–22.9	< 0.001

*Abbreviations: HTN; hypertension. DM; diabetes mellitus. AF; atrial fibrillation. CAD; coronary artery disease. EF; ejection fraction*

## Discussion

The current study aimed to elucidate gender-based disparities in the presentation, management, and outcome of ACS patients in Egypt. This may serve as a model for similar LMICs lacking such data to improve healthcare quality, particularly for women.

Key findings revealed delayed hospital admissions among women after the onset of ACS, mainly because of atypical symptoms. In addition, women received less administration of the potent antiplatelet medication ticagrelor before pPCI, as well as a longer door-to-balloon time.

Women had worse in-hospital outcomes, particularly in terms of heart failure and cardiac mortality. However, short-term 30-day survival against MACE was similar in both genders.

Multivariate regression analysis identified female gender, older age, renal impairment, atrial fibrillation, and ejection EF < 40% as potential predictors for MACE.

Women constituted 29.5% of the total patients in this study, a proportion higher than previously reported in studies discussing ACS in Egypt and nearby Gulf region countries (21% in Egypt vs. 19% in the Gulf region vs. 32% globally), aligning closely with proportions observed in extensive studies conducted in developed countries worldwide [21].

The mean age for ACS patients in this study is 56 years, lower than that reported in developed countries but comparable to figures from third-world nations. This trend may be attributed to the pandemic of diabetes mellitus and smoking in Egypt, potentially associated with metabolic syndrome [22]. Additionally, the clinical profile observed in this study bears similarities to previous findings in Egypt [22]. Consistent with current results, research conducted in HICs and LMICs indicated that women with ACS are typically older than men and have a higher burden of comorbidities such as hypertension and diabetes mellitus [3, 23–26].

Atypical symptoms, more common among women, often go unrecognized as cardiac, leading to delays in management due to socioeconomic factors, literacy levels, and a lack of medical insurance. This delay results in a longer duration from the onset of symptoms to hospital admission, consistent with findings from Sobhy et al. [22], who detected the time elapsed between symptom onset and the initial medical encounter to vary between 30 and 720 min in Egypt. Another study noted that women with ACS tend to report for medical attention later, after symptom onset [4].

Male patients are more frequently prescribed aspirin, ticagrelor, ACEI, and statins compared to females. Jneid et al. [27] observed decreased rates of aspirin administration within the initial 24-hour period among female patients because of non-typical symptoms, leading to delayed recognition of heart attacks in women [28, 29]. It is worth mentioning that female patients with atherosclerotic CVD in a United States study exhibit lower levels of engagement with healthcare providers and underutilization of secondary prevention medications compared to male patients [30].

Among the STEMI subgroup of women who underwent primary PCI (pPCI), door-to-balloon time was longer. This finding matches the studies that revealed that women with STEMI experience primary PCI with longer door-to-balloon delays than males [31].

However, this investigation recorded longer door-to-balloon times than Butala et al. [31], which was 68.91 ± 23.25 min. This may result from different methodologies where Butala et al. collected data from five Egyptian hospitals in Cairo and Alexandria, including two university-affiliated public hospitals, one public hospital affiliated with the Ministry of Health, one private hospital, and one university-affiliated hospital. This confirms the importance of evaluating regional differences, even within the same country.

Regarding revascularization-related interventions, and in contrast to Jneid et al., who noted lower utilization of revascularization procedures among women [27], this research observed no gender differences in the administration of the diagnostic and therapeutic interventions, including primary PCI, consistent with previous results from the Egyptian CardioRisk study [32]. Additionally, all stents used for PCI were drug-eluting stents (DES), in contrast to earlier reports by Sobhy et al. [22], where DES was utilized in only 19.3% of patients and bare metal stents (BMS) in 80.7%.

The overall in-hospital mortality rate was 3.5%, comparable to rates reported in numerous industrialized countries [22]. The in-hospital mortality rate was 2.9%, according to Sobhy et al. [22], and 4.7% in a Greek study [33].

Regarding gender disparities in the prevalence of adverse clinical outcomes, particularly in-hospital cardiac mortality, previous studies have demonstrated conflicting results [4, 34]. While some registries in China [35], Thailand [36], and Malaysia [37] have illustrated no difference in in-hospital mortality, this trial detected poorer in-hospital outcomes for women with worse NYHA classes III and IV and higher rates of in-hospital cardiac mortality.

The higher incidence of in-hospital MACE in women may be attributed to clinical differences. Women with ACS are older at admission, have a greater variety of atypical symptoms and a more significant number of comorbidities, experience delayed admission, are less likely to receive ticagrelor, and have longer door-to-balloon time.

The present findings are partially consistent with previous research by Butala et al., which reported that women in Egypt have a higher incidence of in-hospital mortality. However, unlike the current results, Butala et al. observed a higher 30-day MACE in women [31]. A Middle East and Gulf region study demonstrated higher in-hospital mortality rates in women [37].

A follow-up period of one month did not reveal a statistically significant difference between males and females in terms of unplanned readmissions for heart failure, reinfarction, stroke, and cardiac mortality. The slightly diminished left ventricular EF at discharge (49.56 ± 8.2) and the low rates of unplanned readmissions within 30 days for heart failure and for reinfarction serve as indicators of the effectiveness of the implemented revascularization interventions.

### Study limitations

1. The results are derived from a single center.
2. Individuals who passed away before their hospitalization were not included.
3. No data were collected regarding socioeconomic status, income, or level of education, which could potentially account for the disparities observed in treatment and outcomes.
4. Certain physicians might have faced cultural barriers when confronted with sensitive topics like smoking disclosure among female patients.
5. The study lacked information regarding the duration of each cardiovascular risk factor.

## Conclusions

The current study suggests that women with ACS in Egypt exhibit a higher risk profile for CVD compared to men and tend to present later with atypical symptoms. Women additionally experience poorer in-hospital MACE and higher cardiac mortality. Therefore, increasing awareness about ACS syndrome and eliminating obstacles that delay hospital admission are imperative.

### Electronic supplementary material

Below is the link to the electronic supplementary material.

Supplementary Material 1

Supplementary Material 2

## Data Availability

The data supporting the conclusions of this study are available upon reasonable request from the corresponding author.

## Abbreviations

ACS: Acute coronary syndrome
HICs: High-income countries
PCI: Percutaneous coronary intervention
pPCI: Primary percutaneous coronary intervention
AMI: Acute myocardial infarction
LMICs: Low- and middle-income countries
STEMI: ST-segment elevation myocardial infarction
Non-STEMI: Non-ST-segment elevation myocardial infarction
ECG: Electrocardiogram
EF: Ejection fraction
ESC: European Society of Cardiology
MACE: Major adverse cardiovascular events
MINOCA: Myocardial infarction with no obstructive coronary artery disease
CABG: Coronary artery bypass graft
CVD: Cardiovascular disease
CCU: Coronary care unit
DES: Drug-eluting stents
MI: Myocardial infarction
