# Supplementary material for: Gender differences in presentation, management, and outcomes among Egyptian patients with acute coronary syndrome: a single-centre registry
Source: BMC Cardiovasc Disord. 2024 Jul 16;24:364. doi: 10.1186/s12872-024-03996-8 (PMC11251322; doi:10.1186/s12872-024-03996-8)
Supplement: Supplementary file 1 — Supplementary Material 1 [file 12872_2024_3996_MOESM1_ESM.docx]

Supplementary Tables

**Supplementary Table 1**: Laboratory findings in both genders.

|  | Men | Women | P value | Total |
| --- | --- | --- | --- | --- |
|  | | | | |
| TCL-C (mg/dl) ^a^ | 231.32 ±36.2 | 232.42 ±39.43 | .786* | 231.57 ± 37.15 |
| LDL-C (mg/dl)  ^a^ | 129.49±44.33 | 133.18 ±41.32 | .439* | 130.52 ± 43.47 |
| HDL-C (mg/dl) ^a^ | 47.06±9.87 | 47.07±7.5 | .997* | 47.07 ± 9.23 |
| Creatinine (mg/dl) ^a^ | 1.3±1.29 | 1.28±1.26 | .913* | 1.16 ± 0.33 |
| RBG (mg/dl) ^a^ | 175±73.92 | 194±92.8 | .052* | 181.48 ± 80.29 |
| HBAIC (mg/dl) ^a^ | 6.89± 1.89 | 9.65±20.98 | .268* | 7.02 ± 1.85 |
| On admission Positive Troponin,  n (%) | 239, (84.75) | 107, (90.68) | .114** | 346, (86.5) |

*^a^ Results are expressed as mean ± standard deviation*

**Unpaired t-test;*

*** chi-square test*

*Abbreviation; HDL-C; High-density lipoprotein cholesterol. LDL-C; Low-density lipoprotein cholesterol. TC; total cholesterol. RBG; random blood sugar. HBA1C; glycated haemoglobin*

**Supplementary Table 2**: Pharmacotherapy, coronary angiography and managing strategies between men and women.

| Variable | men | | | women | | | | P**  value | total | |
| --- | --- | --- | --- | --- | --- | --- | --- | --- | --- | --- |
|  | n | % | | n | | | % |  | n | % |
| Pharmacotherapy | | | | | | | | | | |
| Th.th | 39 | | 13.88% | | 9 | 7.56% | | .076 | 48 | 12% |
| Aspirin | 281 | | 99.65% | | 113 | 95.76% | | .004 | 394 | 98.5% |
| Clopidogrel | 189 | | 67.02% | | 83 | 70.34% | | .377 | 272 | 68% |
| Ticagrelor | 91 | | 32.27% | | 27 | 22.88% | | .034 | 118 | 29.5% |
| UFH | 221 | | 78.36% | | 84 | 71.18% | | .124 | 305 | 76.25% |
| LMWH | 38 | | 13.4% | | 22 | 18.64% | | .104 | 60 | 15% |
| Beta-blocker | 211 | | 74.82% | | 82 | 69.49% | | .178 | 293 | 73.25% |
| ACEIs | 187 | | 66.31% | | 54 | 45.76% | | <.001 | 241 | 60.25% |
| ARBs | 52 | | 18.44% | | 21 | 17.8% | | .663 | 73 | 18.25% |
| Statin | 266 | | 94.33% | | 98 | 83.05% | | <.001 | 364 | 91% |
| Non-culprit Coronaries with significant lesions : | | | | | | | | | | |
| LM | 10 | 3.57% | | 4 | | | 3.39% | .928 | 14 | 3.52% |
| LAD | 66 | 23.4% | | 24 | | | 26.67% | .396 | 90 | 22.5% |
| Diagonal | 17 | 6.03% | | 8 | | | 6.78% | .799 | 25 | 6.25% |
| LCX | 44 | 15.6% | | 21 | | | 17.7% | .332 | 65 | 16.25% |
| OM | 10 | 3.55% | | 6 | | | 5.08% | .474 | 16 | 4% |
| RCA | 71 | 25.18% | | 22 | | | 18.64% | .142 | 93 | 23.25% |
| PDA | 4 | 1.42% | | 3 | | | 2.54% | .444 | 7 | 1.75% |
| PL | 1 | 0.35% | | 0 | | | 0% | .791 | 1 | 0.25% |
| IF PCI. number of DES on culprit lesion : | | | | | | | | | | |
| 1 stent | 139 | 49.2% | | 50 | | | 42.3% | 0.771 | 189 | 58.15% |
| 2stents | 58 | 20.5% | | 24 | | | 20.3% |  | 82 | 25.23% |
| 3 stents | 2 | 0.7% | | 3 | | | 2.5% |  | 5 | 1.54% |
| 0 (only balloon angioplasty ) | 15 | 5.3% | | 6 | | | 5% |  | 21 | 5.25% |
| Deferred stenting | 4 | 1.45% | | 1 | | | 0.89% |  | 5 | 1.25% |
| GP IIb/IIIa I | 52 | 18.43% | | 22 | | | 18.6% | .962 | 74 | 18.5% |
| Type of PCI | | | | | | | | | | |
| pPCI | 208 | 73.75% | | 83 | | | 70.3% | .693 | 291 | 72.75% |
| Facilitated PCI | 26 | 9.21% | | 7 | | | 5.9% |  | 33 | 8.25% |
| Rescue PCI | 8 | 2.83% | | 2 | | | 1.7% |  | 10 | 2.5% |
| strategies for further management after treating the culprit lesion: | | | | | | | | | | |
| Further PCI for non-culprit | 172 | 61.7% | | 62 | | | 54.2% | .626 | 234 | 58.5% |
| Medical treatment | 52 | 18.4% | | 35 | | | 29.6% |  | 87 | 21.75% |
| For CABG | 14 | 5.05% | | 5 | | | 4.23% |  | 19 | 4.0% |

*** chi-square test*

*Abbreviations: Th.th; thrombolytic therapy. UFH; unfractionated heparin. LMWH; low molecular Wight heparin; ACIEs; angiotensin-converting enzyme inhibitors. ARBs; angiotensin receptor blockers; SGLT2is; sodium glucose transferase 2 inhibitors. LM; left main artery. LAD. left anterior descending. RCA; right coronary artery. LCX ; left circumflex . OM; obtuse marginal. PL; posterolateral. PDA; posterior descending artery. MINOCA myocardial infarction with non-obstructive coronary arteries. GBIIb/IIIa I; Glycoprotein IIb/IIIa inhibitors. CABG; coronary artery bypass grafting*
