# Supplementary material for: Gender differences in presentation, management, and outcomes among Egyptian patients with acute coronary syndrome: a single-centre registry
Source: BMC Cardiovasc Disord. 2024 Jul 16;24:364. doi: 10.1186/s12872-024-03996-8 (PMC11251322; doi:10.1186/s12872-024-03996-8)
Supplement: Supplementary file 2 — Supplementary Material 2 [file 12872_2024_3996_MOESM2_ESM.docx]

Supplementary Figures


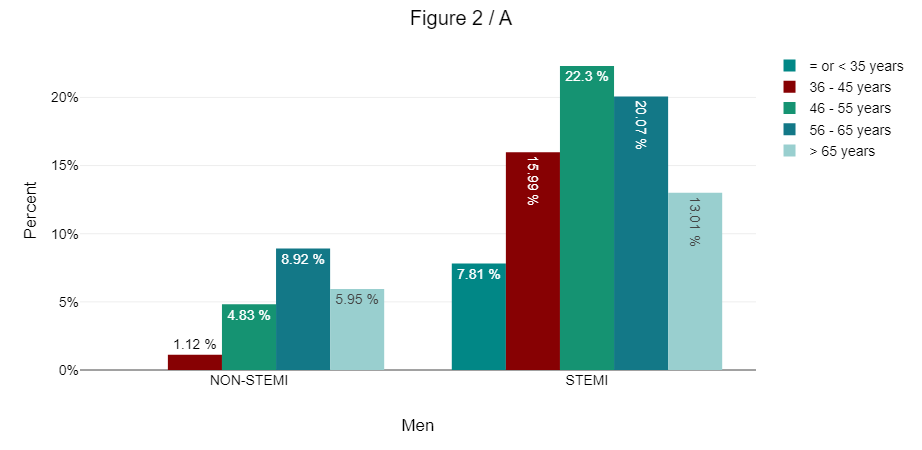


**A -**


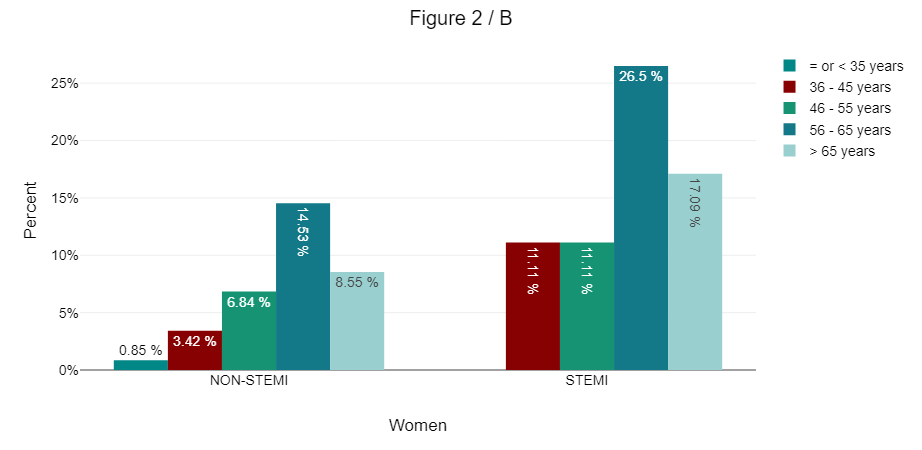


**B -**

- **Supplementary Figure 1:** Age groups among enrolled patients with STEMI and non-STEMI among men (A) and Women (B)


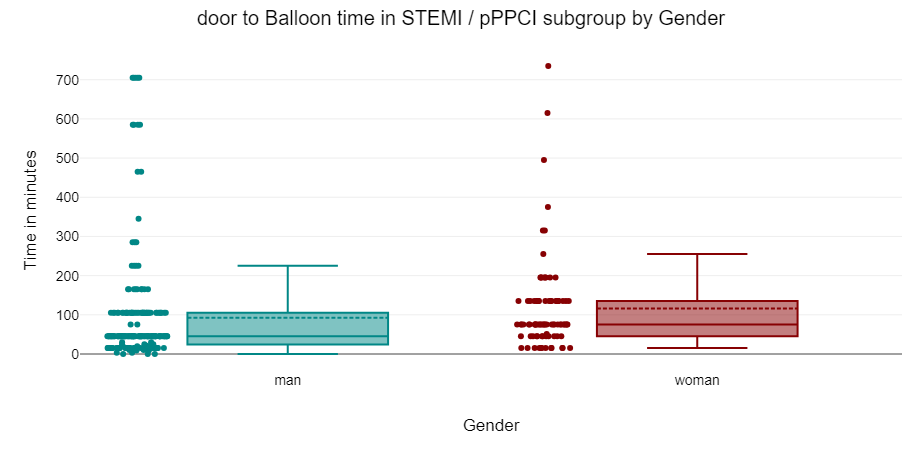


- **Supplementary Figure 2:** Box plots displaying the door-to-balloon times in both genders in STEMI patients who underwent primary PCI subgroup**.**
